# *Porcine circovirus 2* immunology and viral evolution

**DOI:** 10.1186/s40813-015-0012-z

**Published:** 2015-11-19

**Authors:** Tuija Kekarainen, Joaquim Segalés

**Affiliations:** 1 Centre de Recerca en Sanitat Animal (CReSA), Institute of Agrifood Research and Technology (IRTA), Bellaterra, Cerdanyola del Vallès, Spain; 2 Departament de Sanitat i Anatomia Animals, Universitat Autònoma de Barcelona, Bellaterra, Cerdanyola del Vallès, Spain

**Keywords:** *Porcine circovirus 2 *(PCV2), Pig, Immunity, Virus evolution

## Abstract

*Porcine circovirus 2* (PCV2) has and is still causing important economic losses to pig industry. This is due to PCV2-systemic disease (PCV2-SD), formerly known as postweaning multi-systemic wasting syndrome (PMWS), which increases mortality rates and slows down the growth of the animals, as well as other conditions collectively included within the so-called porcine circovirus diseases (PCVD). PCV2-SD affected pigs are considered to be immunosuppressed, with severe lymphocyte depletion and evidence of secondary infections. However, PCV2-infected pigs not developing the disease are able to mount humoral and cellular immune responses and clear the virus or limit the infection. On the contrary, insufficient amounts of neutralizing antibodies have been linked to increased PCV2 replication, severe lymphoid lesions and development of PCV2-SD. Central role in controlling PCV2 infection are played by the antigen specific memory T cells. These cells persist long term post-infection or vaccination and are able to expand rapidly after recall antigen recognition. Most farms in the main pig producing countries are applying vaccination against PCV2 to prevent the disease and improve the farm performance. Vaccines do not induce sterilizing immunity and PCV2 keeps on circulating even in farms applying vaccination. This, together with the high mutation rate of PCV2, world-wide fluctuations in the genotype dominance and emergence of novel genetic variants, warrant close molecular survey of the virus in the field.

## Background

*Porcine circovirus 2*-systemic disease (PCV2-SD), formerly known as postweaning multisystemic wasting syndrome (PMWS), causes important economic losses in commercial pig farms due to increased mortality rates and negative impact on the growth of the animals. Clinical signs of PCV2-SD include wasting or decreased weight gain, anaemia, diarrhoea and/or respiratory distress. Extensive lesions in lymphoid tissues due to depletion of B and T lymphocytes of affected pigs can be observed. Numbers of B- and T-cells are reduced both in blood and lymphoid organs coinciding with the increment of macrophages/monocytes. Detailed description of clinical signs and pathogenesis can be found elsewhere [1]. PCV2 is the necessary but not sufficient etiological agent to trigger PCV2-SD, as well as other porcine circovirus diseases (PCVD). The ubiquitous nature of PCV2 implies that most of the animals worldwide get infected with this virus, but just a proportion of them suffer from overt disease; therefore, most pigs suffer from a PCV2-subclinical infection [2].

Since 2007, a number of commercial vaccines have been launched significantly reducing the economic losses due to PCV2 infection. Despite the vaccines, the virus is still circulating even among vaccinated populations [3]. PCV2 has a circular single-stranded DNA genome of 1.7 kb with limited encoding capacity. Replication associated proteins (Rep and Rep’) are encoded by ORF1, the only structural capsid protein (Cap) is encoded by ORF2, and ORF3 and ORF4 encode for proteins involved in apoptosis and anti-apoptosis, respectively [4]. The *capsid* gene (*cap*) is the most used in phylogenetic studies and four genotypes can be distinguished based on it or complete PCV2 sequences, namely PCV2a, PCV2b, PCV2c and PCV2d [5]. PCV2 is characterized with high mutation rate, genotype dominance shifts and a recent report suggests possible influence of vaccination to its evolution [5–8].

Under ideal conditions, immunological responses against pathogens lead to their clearance and generation of immunological memory. In contrast, pathogen evolution implies to develop strategies to circumvent and exploit the immune system to complete their life cycle. Some pathogens, like PCV2, have the ability to persist in the host, causing a prolonged pro-inflammatory state leading to immunopathological effects. The outcome of PCV2 infection varies greatly between individual pigs and the ones developing a sufficient humoral and cellular immunity clear the infection. However, PCV2-SD affected pigs develop severe lymphocyte depletion [9], which was the first evidence for researchers that immunosuppression was the main feature of the disease. Since then, there has been increasing amount of studies about the involvement of the immune system in the pathogenesis and the strategies PCV2 is using to manipulate the immune cells for its benefit.

### Immunomodulatory capacity of PCV2

Innate, non-adaptive host immune response is the early barrier against pathogen infections. This response is activated immediately after invasion of a new pathogen. If an early response does not lead to clearance of the pathogen, the adaptive immune response takes place. In the process of defence against pathogens, an important role is played by dendritic cells (DC). DCs together with macrophages are first immune cells to encounter pathogens. They are engulfing and digesting pathogens and presenting antigens to T lymphocytes initiating the adaptive immune arm. Two main types of dendritic cells do exist: conventional DC (cDC) with the main role of presenting antigens, and plasmacytoid DC (pDC) which are potent producers of type I interferon (IFN) [10, 11]. As many other viruses, PCV2 is capable of modulating the DC activity. PCV2 is engulfed by DC but does not seem to actively replicate in these cells, neither is affecting their survival [12, 13]. While cDC function is not impaired by PCV2 accumulation, the pDC function is affected by the virus [14, 15]. IFN-α is typically induced by pDCs by many viruses and the best known function of this cytokine is to induce an antiviral state. However, PCV2 is down-regulating the induction of IFN-α in cultured pDCs/monocytic cells, even in the presence of potent IFN-α stimulators [14, 16]. In contrast, in vivo infection with PCV2 has shown to induce IFN-α secretion [17, 18]. Indeed, it has been shown that PCV2 can have both immunostimulatory and inhibitory roles [19–22]. Specifically, it has been demonstrated that the viral genome, or parts of it, can modulate cytokine responses, possibly via inhibitory /stimulatory CpG motifs interacting with cytosolic or endosomal receptors in the cells [19–21]. On the other hand, it has been suggested that the balance between the levels of encapsulated genomic ssDNA (stimulatory effect) and free dsDNA (inhibitory effect) replicative forms of PCV2 determines the immunomodulatory characteristics of PCV2 infection [22].

Another key cytokine which expression PCV2 infection seems to alter, is the tightly regulated, pleiotropic cytokine IL-10. It can be produced by both innate (macrophages, DC) and adaptive cells (B cells and subsets of CD4+ and CD8+ cells). It is able to inhibit the activity of several cell types involved in pathogen clearance like macrophages, Th1 – and NK cells. The importance of IL-10 during PCV2 infection has been studied both in vivo and in vitro. IL-10 expression is induced by PCV2, but not by non-pathogenic PCV1, infection in vitro cultured PBMCs, especially the monocyte/DC/macrophage populations [19, 23]. The release of this cytokine by PCV2 infected PBMCs led to inhibition of IFN-γ, IFN-α and IL-12 stimulated by recall antigen of another virus [19]. Monocyte/DC/macrophage population was shown to be responsible of the IL-10 production and whole non-inactivated virus, but not Cap or Rep proteins, was identified as the triggering viral signal [24]. Therefore, likely pathway for IL-10 induction in this cell population is controlled through Toll-like receptor 9 (TLR9), which is an endosomally expressed receptor recognizing unmethylated DNA [25]. Transcription of IL-10 is also increased in thymus of PCV2-SD affected pigs and this was associated with the thymic depletion and atrophy [26]. Systemic IL-10 secretion has been associated with animals evolving and suffering from PCV2-SD [17, 27]. Indeed, it was suggested that IL-10 induction by PCV2 is a key cytokine leading to immune disorders typically found in PCV2-SD affected pigs. Furthermore, systemic IL-10 levels have been correlated with the viral load in blood at 21 days post-infection [28]. Finally, the source and impact of the IL-10 seen in the in vitro and in vivo studies are supposedly very different considering that these type of studies are conceptually very distinct: in vitro assays allow detailed studies on the early onset of cytokine secretion by identified innate cell types and in vivo studies on the consequences of viral infection on tissues and adaptive immune cells. However, IL-10 is to be considered a cytokine affecting both innate and adaptive immune responses and is one part of the puzzle leading to PCV2-SD.

### Cellular and humoral immune responses against PCV2

Humoral immune responses against PCV2 infection have been studied throughout the pig life from foetuses to adult animals. This is due to the availability of simple methods to measure distinct anti-PCV2 antibodies in easily obtained serum samples and the intensive research done in vaccines, which are based on the immunogenic Capsid protein.

Intra-uterine infection of PCV2 leads to antibody development in foetuses. *In utero* infected foetuses can mount humoral immune responses against PCV2 when they are infected after day 70 of gestation [29, 30]. Lately, it was also shown that maternal antibodies may leak through the placenta and those antibodies can be detected in foetuses without evidence of viral infection during gestation [31]. Indeed, the higher the dam anti-PCV2 antibody titres, the higher the likelihood of antibody detection in its piglets. This finding has a practical consequence about involvement of PCV2 infection in reproductive failure cases, since antibodies can merely be of maternal origin and not a real consequence of a foetal virus infection. Therefore, care should be taken when making conclusions about PCV2 foetal infection based solely on serological tests [31]. However, this finding has not been contrasted so far, and it would be important to generate more data to confirm it.

After birth, piglets are protected against PCV2 infection due to the maternally derived neutralizing antibodies present in the colostrum [32, 33]. These passively acquired antibodies decline during the lactating and nursery periods. Waning of maternal antibodies makes the animals again susceptible to PCV2 infection followed by active seroconversion [34–36]. Neutralizing antibodies are efficient in clearing the virus from circulation and insufficient amounts of neutralizing antibodies have been linked to increased PCV2 replication, severe lymphoid lesions and development of PCV2-SD [37, 38]. Interestingly, during infection, antibodies are produced mainly against the Cap protein but also against non-structural Rep proteins. Anti-cap antibodies are produced earlier and to a higher titre than anti-rep antibodies both in healthy and PCV2-SD affected pigs [39, 40].

Recent studies have taken the cellular immune responses better into account than early studies. This is mainly due to the notion that anti-PCV2 antibodies are not always fully protective and cannot be used to predict protection against PCV-SD [36, 41]. Cellular immunity, especially development of IFN-γ secreting cells (SC), has been inversely correlated with PCV2 viral loads in serum [37, 42, 43]. Since PCV2 specific IFN-γ-SCs are T-cells are able to produce IFN-γ upon stimulation with a recall antigen, their assessment is used to measure the cell mediated immunity and have been shown to increase after PCV2 infection and vaccination [18, 44]. Interestingly, IFN-γ-SCs are specific for both non-structural *Rep* and structural *Cap* proteins [24]. PCV2-SD diseased pigs suffer from B and T cell lymphopenia which is induced by PCV2 infection. Most notably, B and CD3 + CD4 + CD8+ memory/activated Th lymphocytes are depleted and this depletion is related to the development of PCV2-SD [45]. Both vaccination and infection elicit memory/activated Th cells [44, 46]. These cells are activated, antigen specific T cells and memory cells. Antigen specific memory T cells persist long term post-infection/vaccination and are able to expand rapidly after recall antigen recognition, thus being able to prevent PCV2 infection quickly [46]. More specifically, it has been suggested that PCV2 specific IFN-γ/TNF-α co-producing CD4^+^ cells, which are produced upon vaccination/infection, play a central role in controlling and clearing PCV2 infection [44]. Interestingly, this subset of cells was induced in all PCV2 vaccinated/infected animals, while specific antibodies were only detected in nearly 45 % of these pigs [44]. Those results further emphasize the importance of cellular immunity in the control and clearance of PCV2 in infected animals.

### PCV2 evolution

Since the last 20 years, PCV2 is one of the most important swine pathogens. Currently, vaccines against PCV2 are widely used in commercial farms but since the vaccines do not induce sterilizing immunity, the virus keeps circulating even in farms applying vaccination [3, 47]. This is further emphasized by the fact that transmission of PCV2 among vaccinated pigs has an estimated reproduction ratio (R_0_) of 1.5 versus 5.2 in non-vaccinated pigs [48]. Since R_0_ measures the average of secondary infections caused by an individual during its infectious period, values of R_0_ > 1 represents that the pathogen will be maintained within the population. Therefore, PCV2 vaccination is very efficient in decreasing infectious pressure, but not able to clear the virus out from the farm.

Vaccines are based on the Cap protein or whole viruses. In the phylogenetic analysis cap gene is used to classify PCV2 stains into four genotypes. The genetic distance nowadays used to divide PCV2 into genotypes is >3.5 % [49]. Until 2000 PCV2a was the most prevalent genotype found in the field, but was replaced by the PCV2b genotype coinciding with the most severe outbreaks of PCV2-SD [8, 50, 51]. PCV2c was originally found in Denmark and its frequency in field is very low [5, 52]. Lately, PCV2d has emerged and it has been suggested that a new shift in dominant genotype may be on-going [5, 7, 53].

Origin of PCV2 is likely to be relatively recent, about 100 years ago [54]. It has been estimated that PCV2b and PCV2d originated about 20 years ago, timing which would fit to the worldwide emergence of PCV2-SD [5, 54]. Within-genotype genetic divergence reflects the time different genotypes have been circulating in the pig population, PCV2a displaying the highest divergence, followed by PCV2b and PCV2d [5]. Whole genomes between genotypes differ from approximately 8 % to 12 %, PCV2c being the most distinct and followed by PCV2d [5]. Different genotypes’ Capsid protein amino acid sequences differ from 6 % to 15 %.

The variability of PCV2 is created by the high evolutionary rates being 1.2x10^−3^ substitution/site/year, resembling the one from RNA viruses, and recombination [54–58]. Selection which works on the variants is further shaping the evolution of PCV2. Indeed, due to mutations in the *cap* gene, new antigenic variants have emerged [59–61]. Furthermore, applying next-generation-sequencing technology, low frequency mutations have been identified [6]. Low frequency mutants are unfit in the given environment but form reservoirs allowing fast adaptation of the virus in changing environments. On the other hand, sometimes unexpected, beneficial mutations may occur and quickly increase their frequencies in virus population [6].

The *rep* gene is highly conserved while the *cap* gene contains more variability. It has been shown that polyclonal sera from individual pigs can have different titres of neutralizing antibodies against isolates from the same genotype [62]. Indeed, even single mutations may lead to antigenically distinct viruses as shown by studies using monoclonal antibodies to subtype PCV2 strains [63, 64]. These results are not surprising for a virus like PCV2 since it has high evolution rate and infection is highly prevalent under field conditions. However, it is to bear in mind that biological differences, like neutralization by antibodies, are not correlated with the virus sequence but rather depend on the specific virus isolate. Several linear (within the residues 25–43, 69–83, 113–127, 117–131, 169–183 and 193–207) and conformational (within residues 47–85, 165–200 and 230–233) epitope domains have been identified in the Cap protein [64–66]. It has been shown that these regions contain positions which are under negative and positive selection. Especially, non-conserved positions were found to be under positive selection, which can be considered sites targeted by host immune system [59, 67]. Conserved domains in PCV2 proteins are essential sites for their functionality and any mutation in these sites are non-viable for the virus.

One of the current challenges is to understand the long-term effect of vaccination on PCV2 evolution. Theoretically, there are several aspects which suggest that current vaccines could shape the evolution of PCV2. Vaccination is widely applied, covering more than 90 % of the animals in important pig producing countries like USA, Germany and Spain. Pig densities in herds are high, therefore allowing quick spread of any infectious agent. Furthermore, PCV2 is circulating despite vaccination, since vaccines do not prevent PCV2 infection. Indeed, it has been already shown that PCV2 populations found in vaccinating and non-vaccinating farms differ in its genomic composition [6]. Also, emerging PCV2d isolates have been found in vaccinated pigs suffering from PCV2-SD. However, in experimental infection it was shown that current vaccine based on PCV2a is effective against PCV2d [68]. More work is needed to study the effect of vaccination in virus evolution; especially in the cases of disease appearance in vaccinated animals, since it is unclear if vaccination or vaccine has failed (vaccination vs. vaccine failure).

## Conclusions

It is known that most of the pigs mount effective immune responses to clear or limit PCV2 infection. However, a proportion of pigs may not be able to counteract the infection and develop disease. Nowadays, the disease burden can be controlled by efficient vaccination. Vaccination is priming the immune system and allows its quick and effective activation when encountering PCV2. Since vaccines are not able to completely avoid PCV2 infection, and considering the mutation capacity of the virus, further phylogenetic evolution of PCV2 in the vaccination scenario is guaranteed.
